# Effect of a Smartphone App on Weight Change and Metabolic Outcomes in Asian Adults With Type 2 Diabetes: A Randomized Clinical Trial

**DOI:** 10.1001/jamanetworkopen.2021.12417

**Published:** 2021-06-03

**Authors:** Su Lin Lim, Kai Wen Ong, Jolyn Johal, Chad Yixian Han, Qai Ven Yap, Yiong Huak Chan, Yu Chung Chooi, Zhi Peng Zhang, Cheryl Christine Chandra, Anandan Gerard Thiagarajah, Chin Meng Khoo

**Affiliations:** 1 Department of Dietetics, National University Hospital, Singapore; 2 Faculty of Health Sciences, University of Adelaide, Australia; 3 Caring Future Institute, College of Nursing and Health Sciences, Flinders University, Australia; 4 Biostatistics Unit, Yong Loo Lin School of Medicine, National University Health System, Singapore; 5 Singapore Institute for Clinical Services, Agency for Science, Technology and Research, Singapore; 6 National University Polyclinics, Singapore; 7 Division of Endocrinology, National University Hospital, Singapore; 8 Yong Loo Lin School of Medicine, National University of Singapore, Singapore

## Abstract

**Importance:**

Lifestyle interventions are effective in diabetes management, with smartphone apps that manage health data and dietary and exercise schedules gaining popularity. However, limited evidence from randomized clinical trials exists regarding the effectiveness of smartphone-based interventions among Asian adults with type 2 diabetes.

**Objective:**

To compare the effects of a culturally contextualized smartphone-based intervention with usual care on weight and metabolic outcomes.

**Design, Setting, and Participants:**

This randomized clinical trial conducted at multiple primary care centers in Singapore included 305 adults with type 2 diabetes and body mass index (BMI) of 23 or greater who had literacy in English and smartphone access. Participants were recruited between October 3, 2017, and September 9, 2019, and were randomly assigned (1:1; stratified by gender, age, and BMI) to intervention (99 participants) or control (105 participants) groups. Participants’ data were analyzed using intention-to-treat analysis.

**Interventions:**

Both control and intervention participants received diet and physical activity advice from a dietitian at a baseline face-to-face visit. Intervention participants additionally used a smartphone app to track weight, diet, physical activity, and blood glucose and then communicated with dietitians for 6 months.

**Main Outcomes and Measures:**

Primary outcome was change in body weight, while secondary outcomes were changes in hemoglobin A_1c_ (HbA_1c_), fasting blood glucose, blood pressure, lipids, and diet. Post hoc analyses included glycemic changes in the subgroup with HbA_1c_ levels of 8% or greater and diabetes medication changes.

**Results:**

Among the 204 randomized participants (mean [SD] age, 51.2 [9.7] years; 132 [64.7%] men), baseline mean (SD) BMI was 30.6 (4.3). Compared with the control group, intervention participants achieved significantly greater reductions in weight (mean [SD] change, −3.6 [4.7] kg vs −1.2 [3.6] kg) and HbA_1c_ levels (mean [SD] change, −0.7% [1.2] vs −0.3% [1.0]), with a greater proportion having a reduction in diabetes medications (17 participants [23.3%] vs 4 participants [5.4%]) at 6 months. The intervention led to a greater HbA_1c_ reduction among participants with HbA_1c_ levels of 8% or higher (mean [SD] change, −1.8% [1.4] vs −1.0% [1.4]; *P* = .001). Intergroup differences favoring the intervention were also noted for fasting blood glucose, diastolic blood pressure, and dietary changes.

**Conclusions and Relevance:**

In this study, a smartphone-based lifestyle intervention was more effective in achieving weight and glycemic reductions among Asian adults with type 2 diabetes compared with usual care, supporting the use of apps in lifestyle intervention delivery.

**Trial Registration:**

anzctr.org.au Identifier: ACTRN12617001112358

## Key Points

**Question:**

What is the effect of a culturally contextualized smartphone-based lifestyle intervention on weight change and metabolic outcomes in Asian adults who have overweight or obesity and type 2 diabetes compared with usual care?

**Findings:**

In this randomized clinical trial of 204 adults with type 2 diabetes, the use of a smartphone app tracking personal health data and using integrated behavioral modification strategies led to significantly greater reductions in weight and hemoglobin A_1c_, along with a significantly greater proportion of patients with a reduction in diabetes medication dosages compared with usual care at 6 months.

**Meaning:**

These findings suggest that a mobile health lifestyle intervention has the potential to improve weight and glycemic outcomes among individuals who have overweight or obesity in an Asian population with type 2 diabetes.

## Teaser

This randomized clinical trial of adults in Singapore with type 2 diabetes and overweight or obesity assessed whether use of a smartphone app with behavior modification strategies affected weight change and metabolic outcomes.

## Introduction

Lifestyle interventions delivered by health care professionals to promote weight loss are recommended as a key treatment in type 2 diabetes management.^1,2,3^ Medical nutrition therapy, in particular, improves weight and metabolic outcomes.^4,5,6^ Weight loss in turn improves insulin resistance associated with diabetes-related metabolic disorders,^3,7^ with glycemic improvements observed at 3% weight loss,^8,9^ and is particularly relevant in Asian populations with increasing rates of obesity.^10,11^ Traditionally, lifestyle interventions involve multiple face-to-face sessions, which tend to be labor-intensive and require facilities planning.^12,13^ Studies have shown that travel distances, time constraints, and costs are factors detracting from the effectiveness of lifestyle interventions, with drop-out rates as high as 35%.^14,15^

In recent years, smartphone apps have been gaining popularity in the delivery of lifestyle interventions in chronic disease management, owing to the ability to circumvent these issues.^16,17,18,19,20^ To improve the acceptability, adherence, and effectiveness of interventions, tailoring app content to the cultural norms and values of users is recommended.^21,22,23^ However, only a small number of randomized clinical trials (RCTs) have investigated the effect of culturally contextualized smartphone-based interventions on weight loss in Asian populations with type 2 diabetes.^24,25,26,27,28^

Nutritionist Buddy Diabetes is a locally contextualized mobile app that integrates behavioral treatment, evidence-based diabetes management strategies,^29,30,31^ and dietitian support to promote weight and glycemic control. (eAppendix, eFigure 1, and eFigure 2 in Supplement 1 provide further description and a full list of features.) The app includes a local food database and an algorithm that generates healthier food alternatives based on the cuisines of foods keyed in by users, which is especially pertinent in multicultural Singapore. Educational videos available in the app were developed locally. The app also offers support from local dietitians familiar with cultural practices and festivities of local ethnic groups, who are able to consider culturally specific notions of stigma and provide recommendations in line with the cultural norms of users. In a 2020 RCT,^32^ the app was found to significantly reduce body weight among patients with nonalcoholic fatty liver disease. In this trial, the Diabetes Lifestyle Intervention using Technology Empowerment (D’LITE) study, we compared the effectiveness of a weight loss lifestyle intervention delivered using the app via in-app coaching by dietitians with usual care, focusing on body weight change and metabolic profiles among Asian patients with type 2 diabetes and overweight or obesity who were not receiving insulin.

## Methods

### Study Design

The D’LITE study was a parallel multicenter 2-group RCT (protocol available in Supplement 2). Follow-up at 1 year and 2 years is ongoing; this article presents results from the first 6 months of study. The study was conducted in accordance with the Declaration of Helsinki,^33^ and received ethical approval from the National Healthcare Group Domain Specific Review Board in Singapore. All participants provided written consent prior to study participation. The trial was prospectively registered at the Australian New Zealand Clinical Trials Registry.

### Participants and Eligibility Criteria

Participants were recruited between October 2017 and September 2019 from health screening facilities by research staff. To facilitate enrollment, recruitment was extended to include government polyclinics, general practitioner clinics, and hospital outpatient clinics in Singapore. Inclusion criteria included the presence of physician-diagnosed type 2 diabetes, age between 21 to 75 years, body mass index (BMI; calculated as weight in kilograms divided by height in meters squared) 23.0 or greater, literacy in English, and smartphone access. Participants with heart failure, advanced kidney disease, type 1 diabetes, severe cognitive or psychological disabilities, depression, untreated hypothyroidism, thalassemia, or blood disorders or who were pregnant were excluded from the study. Early in recruitment, participants with insulin use were excluded because of concerns over hypoglycemia risk, as the study did not provide services for the active titration of diabetes therapy as intervention progressed. The decision was also made to exclude participants with untreated anemia or medication noncompliance to minimize confounding factors on glycemic outcomes.

### Randomization and Masking

Eligible participants were randomized to either the control or intervention group in a 1:1 allocation ratio via block randomization stratified by gender, BMI (<27.5 or ≥27.5), and age (<50 years or ≥50 years), which was changed from a previous stratification (at 40 years) 2 months postrecruitment due to a noticeably larger number of older participants. Participants were allocated to either group by drawing personally from sealed, stratified opaque envelopes, each containing an equal proportion of intervention and control group assignments. To ensure high-quality envelope concealment, third-party personnel not involved in the study prepared the envelopes before the commencement of recruitment using matched block methods. Randomization was performed at 3 government polyclinics and 1 hospital outpatient clinic, which the research team visited on a rotational basis. Masking of participants and dietitians was not possible following group allocation because of the nature of the intervention.

### Interventions

All control and intervention participants received a single 45- to 60-minute advisory session from a registered research dietitian concerning diet and physical activity, as per American Dietetic Association (ADA) guidelines,^34^ at baseline. All participants were issued a standardized digital weighing scale (Omron Healthcare) and continued to receive standard diabetes care from their usual health care professionals.

Participants assigned to the intervention group were required to use the app for 6 months to track weight twice weekly and diet and physical activity daily, and to communicate regularly with the research dietitians via the app. Intervention participants chose a weight loss goal of 3% to 10%, depending on individual preferences, and were encouraged to achieve individualized calorie and carbohydrate goals and an activity goal of 10 000 steps daily set by the app. They were also provided with a glucometer (Abbott Laboratories) to track fasting and postprandial blood glucose 2 days weekly. Educational videos lasting approximately 3 minutes each were pushed to the participants weekly via the app in the first 3 months. The 2 dietitians on the research team (K.W.O. and J.J.) supported the participants by messaging them via the app every few days in the first 3 months, and weekly in the subsequent 3 months, spending 1 to 15 minutes on each participant each time. They regularly reviewed goals with intervention participants, provided individualized feedback, and used the usual motivational techniques to guide participants in making lifestyle changes, including helping them to identify and cope with barriers and to use prompts and cues.^35^

### Outcomes

The primary outcome was the change in body weight 6 months postintervention, while secondary outcomes were changes in body weight 3 months postintervention, metabolic profiles (including hemoglobin A_1c_ [HbA_1c_], fasting blood glucose [FBG], blood pressure, total cholesterol, triglycerides, and low-density and high-density lipoprotein levels), creatinine levels, and dietary intake. In the post hoc analyses, changes in physical activity were included as a secondary outcome, along with changes in HbA_1c_ and FBG levels for the subgroup with suboptimal diabetes control (ie, HbA_1c_ levels ≥8%) and changes in dosages of diabetes medication for the subgroup using diabetes medications.

During the study visits at the clinic, research staff measured participants’ body weight using a calibrated digital weighing scale (Omron Healthcare) and blood pressure via an automatic blood pressure monitor (Omron Healthcare). Blood samples were obtained after an overnight fast to determine HbA_1c_, FBG, total cholesterol, triglycerides, and low-density and high-density lipoprotein levels and sent for testing at the National University Hospital Referral Laboratory or National Healthcare Group Diagnostics (both accredited by the College of American Pathologists). Laboratory technicians were masked to group allocation.

Two-day food diaries were collected at baseline, 3 months, and 6 months to evaluate energy and macronutrient intakes. A registered dietitian analyzed dietary intake using the app’s nutrient analysis platform, which utilizes Singapore Energy and Nutrient Composition of Food, Malaysian Food Composition, and US Department of Agriculture food databases, along with nutritional information from food packaging and nutrient analysis of recipes. Data on participants’ diabetes medications and physical activity were collected at baseline and during outcome visits via survey questions regarding changes in the dosages of diabetes medications and the total time spent exercising per week. Medication changes were made at the discretion of participants’ own physicians, with medication costs derived from the Pharmaceutical Society of Singapore Database and the private rates charged by the National University Hospital, Singapore.

### Sample Size

The sample size was calculated based on the assumption of at least a moderate Cohen effect size of 0.5 for the difference in weight loss between groups at 6 months postrandomization. A minimum sample size of 85 participants per group would provide 90% power at .05 level of significance in 2-sided tests. A total sample size of 190 participants (95 per group) was planned, factoring a 10% attrition rate.

### Statistical Analysis

All analyses were performed using SPSS for Windows version 25.0 (IBM Corp). Continuous variables were presented as means with standard deviations, and categorical variables as frequencies and percentages. Parametric tests were used where normality and homogeneity assumptions were satisfied, otherwise Mann-Whitney *U* tests were performed. Generalized linear mixed model analysis was performed on the change from baseline for each numerical outcome to account for clustering effect of recruitment sources as random effect. Subgroup analysis on participants with suboptimal baseline HbA_1c_ levels (ie, ≥8%) was performed on changes in HbA_1c_ and FBG. Comparison of changes from baseline between control and intervention groups was performed using a paired *t* test. Type I error for multiple comparisons was adjusted using the Benjamini-Hochberg procedure with false discovery rate at 0.20. Generalized Poisson mixed-model analysis was performed for changes in medication dosages of subgroup taking diabetes medications, with relative risks presented. Statistical significance was set at 2-sided *P* < .05. Between-group Cohen *d* effect sizes were calculated. Multiple imputation methods^36^ were used to derive missing data points, with 5 imputations performed for each missing value using the Markov chain Monte Carlo method with predictive mean matching for the primary outcome, secondary outcomes, randomization group, and demographic characteristics. Results from 5 imputed data sets were combined.

## Results

### Participants

A total of 305 participants were screened, with 204 participants enrolled and randomized to the control (105 participants) or intervention (99 participants) groups. Nine participants (4 control and 5 intervention) withdrew from the study (Figure). At baseline, participants had a mean (SD) age of 51.2 (9.7) years, BMI of 30.6 (4.3), and HbA_1c_ levels of 7.4% (1.3); 132 participants were men (64.7%) (Table 1). Baseline characteristics were similar between study groups, except for a significantly higher diastolic blood pressure in the control group.

**Figure.**
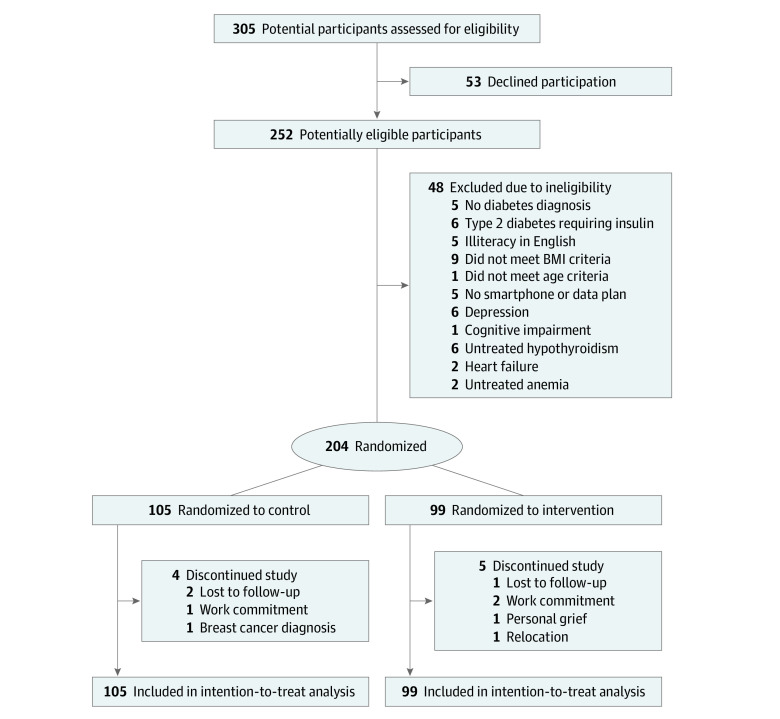
Participant Flowchart

**Table 1.** Baseline Characteristics of Study Participants

Characteristic	Participants, mean (SD)	
	Control group (n = 105)	Intervention group (n = 99)
Gender, No. (%)		
Men	66 (62.9)	66 (66.7)
Women	39 (37.1)	33 (33.3)
Ethnicity, No. (%)		
Chinese	66 (62.9)	66 (66.7)
Malay	20 (19)	18 (18.2)
Indian	18 (17.1)	11 (11.1)
Other	1 (1)	4 (4)
Age, y		
Mean	50.8 (10.0)	51.6 (9.4)
Range	22-72	22-68
Weight, kg	85.6 (15.9)	84.0 (12.6)
BMI	30.9 (4.5)	30.3 (4.0)
HbA_1c_, %	7.5 (1.3)	7.4 (1.2)
Fasting blood glucose, mg/dL	146.0 (43.2)	146.0 (37.8)
Systolic blood pressure, mm Hg	135.3 (13.0)	134.7 (13.5)
Diastolic blood pressure, mm Hg	85.7 (9.8)	82.7 (8.8)
Total cholesterol, mg/dL	183.4 (40.9)	178.8 (35.9)
LDL cholesterol, mg/dL	105.8 (34.8)	103.1 (32.4)
HDL cholesterol, mg/dL	46.7 (9.7)	46.0 (9.3)
Triglycerides, mg/dL	163.7 (95.6)	152.2 (65.5)
Creatinine, μmol/L	73.3 (17.8)	73.2 (17.5)
Length of diabetes condition, y	4.2 (3.6)	5.2 (4.5)
Diabetes treatment, No. (%)		
Diet only	33 (31.4)	26 (26.3)
Oral medication	72 (68.6)	73 (73.7)
Comorbidity, No. (%)		
Hypertension	72 (68.6)	67 (67.7)
Hyperlipidemia	71 (67.6)	72 (72.7)
Others	2 (1.9)	7 (7.1)
Annual cost of diabetes medications, $	667.4 (869.3)	785.9 (822.5)
Nutrient intake		
Calorie, kcal/d	1807.8 (500.0)	1855.5 (545.8)
Carbohydrate, g/d	211.9 (62.8)	213.5 (63.0)
Sugar, g/d	53.5 (25.9)	54.8 (29.8)
Protein, g/d	77.6 (24.0)	79.7 (25.6)
Total fat, g/d	71.6 (23.1)	75.9 (32.0)
Saturated fat, g/d	28.4 (10.9)	29.4 (13.9)
Fiber, g/d	17.5 (6.2)	17.9 (6.0)
Physical activity, min/wk	88.0 (122.9)	102.0 (112.4)

Abbreviations: BMI, body mass index (calculated as weight in kilograms divided by height in meters squared); HbA_1c_, hemoglobin A_1c_; HDL, high-density lipoprotein; LDL, low-density lipoprotein.

SI conversion factors: To convert creatinine to micromoles per liter, multiply by 88.4; glucose to millimoles per liter, multiply by 0.0555; HbA_1c_ to proportion of total hemoglobin, multiply by 0.01; total, HDL, and LDL cholesterol to millimoles per liter, multiply by 0.0259; triglycerides to millimoles per liter, multiply by 0.0113.

Participants’ data were analyzed using intention-to-treat analysis. Complete outcome data were available for 94.6% of all participants at 6 months as detailed in eTables 1 to 3 in Supplement 1. The Little test showed that the data were consistent with the assumption that they were missing completely at random (*P* = .08).

### Body Weight

Table 2 shows changes in weight and metabolic parameters between groups. At 6 months, participants in the intervention group achieved significantly greater reduction in body weight compared with the control group (mean [SD] weight change, −3.6 [4.7] kg vs −1.2 [3.6] kg; *P* < .001). Between group difference in weight loss showed a moderate Cohen effect size of 0.57. This corresponded to a significantly greater percentage of weight loss in the intervention group compared with the control group (−4.3% [5.4] vs −1.4% [4.2]; *P* < .001)

**Table 2.** Primary and Secondary Outcomes at 3 and 6 Months After Enrollment Using Intention-to-Treat Analysis^a^

Outcome variable	Participants, No.	Mean (SD) change from baseline		Between-group difference		
		Control	Intervention	Mean difference (95% CI)	*P* value	Cohen *d*
**Weight and glycemic control (control group, 105 participants; intervention group, 99 participants)**						
Change in weight, kg						
3 mo	204	−0.6 (2.7)^b^	−3.0 (3.8)^b^	−2.3 (–3.2 to–1.4)	<.001	0.73
6 mo	204	−1.2 (3.6)^b^	−3.6 (4.7)^b^	−2.4 (−3.5 to −1.3)	<.001	0.57
Change in weight, %						
3 mo	204	−0.8 (3.2)	−3.5 (4.4)	−2.8 (−3.9 to −1.7)	<.001	0.7
6 mo	204	−1.4 (4.2)	−4.3 (5.4)	−2.9 (−4.2 to −1.6)	<.001	0.6
Change in BMI						
3 mo	204	−0.2 (1.0)^b^	−1.1 (1.3)^b^	−0.8 (−1.2 to −0.5)	<.001	0.78
6 mo	204	−0.4 (1.3)^b^	−1.3 (1.7)^b^	−0.9 (−1.3 to −0.5)	<.001	0.59
Change in HbA_1c_, %						
3 mo	204	−0.3 (1.0)^b^	−0.7 (1.1)^b^	−0.4 (−0.7 to −0.1)	.006	0.38
6 mo	204	−0.3 (1.0)^b^	−0.7 (1.2)^b^	−0.4 (−0.7 to −0.1)	.01	0.36
Change in fasting blood glucose, mg/dL						
3 mo	204	−3.6 (32.4)	−18.0 (37.8)^b^	−14.4 (−23.4 to −3.6)	.005	0.41
6 mo	204	−1.8 (25.2)	−14.4 (37.8)^b^	−12.6 (−23.4 to −3.6)	.01	0.39
**Blood pressure (control group, 72 participants; intervention group, 67 participants)**						
Change in systolic blood pressure, mm Hg						
3 mo	139	−2.2 (14.7)	−6.5 (13.1)^b^	−4.2 (−8.8 to 0.3)	.07	0.31
6 mo	139	−5.0 (13.8)^b^	−7.8 (15.2)^b^	−2.8 (−7.7 to 2.0)	.25	0.19
Change in diastolic blood pressure, mm Hg						
3 mo	139	−1.8 (11.0)	−4.4 (9.9)^b^	−2.6 (−6.2 to 0.9)	.15	0.25
6 mo	139	−3.1 (9.2)^b^	−5.4 (11.1)^b^	−2.4 (−5.7 to 1.0)	.17	0.23
**Cost of diabetes medications (control group, 74 participants; intervention group, 73 participants)**						
Change in annual cost, $						
3 mo	147	13.1 (123.6)	−56.9 (278.3)	−70.0 (−137.2 to −2.8)	.04	0.33
6 mo	147	85.7 (313.3)^b^	−59.7 (387.9)	−145.3 (−252.4 to −38.3)	.01	0.41
**Lipids (control group, 71 participants; intervention group, 72 participants)**						
Change in total cholesterol, mg/dL						
3 mo	143	−2.32 (33.2)	−12.4 (25.5)^b^	−10.4 (−19.7 to −0.4)	.04	0.34
6 mo	143	−5.8 (42.9)	−9.3 (37.1)^b^	−3.1 (−16.2 to 10.0)	.65	0.09
Change in LDL cholesterol, mg/dL						
3 mo	143	−1.2 (27.0)	−7.7 (22.4)^b^	−6.2 (−14.7 to 1.9)	.14	0.26
6 mo	143	−3.1 (33.2)	−6.6 (33.2)	−3.5 (−13.9 to 7.3)	.53	0.1
Change in HDL cholesterol mg/dL						
3 mo	143	1.2 (8.9)	1.2 (10.4)	0.4 (−2.7 to 3.1)	.90	0.00
6 mo	143	1.2 (8.9)	1.5 (9.7)	0.4 (−2.7 to 3.9)	.76	0.04
Change in triglycerides, mg/L						
3 mo	143	−22.1 (103.5)	−31.9 (57.5)^b^	−9.7 (−37.2 to 17.7)	.48	0.12
6 mo	143	−31.9 (102.7)^b^	−22.1 (67.3)^b^	9.7 (−18.6 to 38.9)	0.49	0.11
Change in creatinine, μmol/L						
3 mo	204	0 (10.1)	−0.2 (8.1)	−0.1 (−2.6 to 2.4)	.91	0.02
6 mo	204	1.6 (9.7)	0.9 (9.2)	−0.6 (−3.2 to 1.9)	.63	0.07
**Other dietary and physical activity variables (control group, 105 participants; intervention group, 99 participants)**						
Change in calorie, kcal/d						
3 mo	204	−212.9 (566.0)^b^	−583.3 (571.4)^b^	−370.5 (−527.0 to −214.0)	<.001	0.65
6 mo	204	−245.8 (466.7)^b^	−551.3 (515.4)^b^	−305.8 (−441.0 to −170.7)	<.001	0.62
Change in carbohydrate, g/d						
3 mo	204	−25.8 (64.0)^b^	−65.5 (72.6)^b^	−39.7 (−58.5 to −20.8)	<.001	0.58
6 mo	204	−28.9 (64.5)^b^	−64.4 (64.5)^b^	−35.5 (−53.4 to −17.6)	<.001	0.55
Change in sugar, g/d						
3 mo	204	−9.6 (34.5)^b^	−22.0 (33.2)^b^	−12.4 (−21.8 to −3.1)	.009	0.37
6 mo	204	−9.8 (33.2)^b^	−21.5 (29.7)^b^	−11.7 (−20.7 to −2.7)	.01	0.37
Change in protein, g/d						
3 mo	204	−5.2 (29.8)	−16.5 (29.6)^b^	−11.3 (−19.4 to −3.3)	.006	0.38
6 mo	204	−8.2 (28.5)^b^	−14.7(26.3)^b^	−6.5 (−14.0 to 1.1)	.09	0.24
Change in total fat, g/d						
3 mo	204	−7.4 (30.7)^b^	−28.7 (34.1)^b^	−21.4 (−30.3 to −12.5)	<.001	0.66
6 mo	204	−9.9 (22.7)^b^	−26.5 (33.1)^b^	−16.6 (−24.3 to −8.8)	<.001	0.58
Change in saturated fat, g/d						
3 mo	204	−4.0 (14.0)^b^	−11.8 (14.1)^b^	−7.9 (−11.8 to −4.0)	<.001	0.56
6 mo	204	−4.4 (11.2)^b^	−11.9 (14.1)^b^	−7.5 (−11.0 to −4.0)	<.001	0.59
Change in fiber, g/d						
3 mo	204	−0.8 (8.1)	−3.5 (7.6)^b^	−2.7 (−4.8 to −0.5)	.02	0.34
6 mo	204	−2.2 (7.2)^b^	–2.7 (8.0)^b^	−0.6 (−2.6 to 1.4)	.54	0.07
Change in physical activity, min/wk						
3 mo	204	14.4 (103.4)	67.9 (173.4)^b^	53.4 (14.9 to 91.9)	.007	0.37
6 mo	204	9.0 (122.0)	71.4 (207.9)^b^	62.4 (16.1 to 108.6)	.009	0.37

Abbreviations: BMI, body mass index (calculated as weight in kilograms divided by height in meters squared); HbA_1c_, hemoglobin A_1c_; HDL, high-density lipoprotein; LDL, low-density lipoprotein.

SI conversion factors: To convert creatinine to micromoles per liter, multiply by 88.4; glucose to millimoles per liter, multiply by 0.0555; HbA_1c_ to proportion of total hemoglobin, multiply by 0.01; total, HDL, and LDL cholesterol to millimoles per liter, multiply by 0.0259; triglycerides to millimoles per liter, multiply by 0.0113.

^a^ Results are presented for imputed data using the Markov chain Monte Carlo method.

^b^ Statistically significant change from baseline to postintervention at *P* < .05, after Benjamini-Hochberg correction with false discovery rate at 0.20 and with 76 participants.

### Metabolic Outcomes

Mean (SD) HbA_1c_ levels improved by 0.7% (1.2) (to convert to proportion of total hemoglobin, multiply by 0.01) and 0.3% (1.0) in the intervention and control groups, respectively, at 3 months and 6 months. Mean fasting blood glucose improved by 14.4 (37.8) mg/dL (to convert to millimoles per liter, multiply by 0.0555) and 1.8 (25.2) mg/dL in the intervention and control groups respectively (Table 2). Post hoc analysis of the HbA_1c_ subgroup (55 participants) revealed greater improvements in HbA_1c_ in the intervention group among patients with baseline HbA_1c_ levels of 8% or higher (mean [SD] change, −1.8% [1.4] vs −1.0% [1.4]; *P* = .02) (Table 3). Between-group differences in blood pressure and lipids were not observed at 6 months.

**Table 3.** Changes in HbA_1c_ and Fasting Blood Glucose at 3 and 6 Months of Intervention for HbA_1c_ Subgroups^a^

Outcome variable	Participants, No.	Mean (SD) change from baseline		Between-group difference		
		Control	Intervention	Mean difference (95% CI)	*P* value	Cohen *d*
**HbA_1c_ ≥8% subgroup**^**a**^** (control group, 29 participants; intervention group, 26 participants)**						
Change in HbA_1c_, %						
3 mo	55	−1.1 (1.3)^b^	−1.8 (1.4)^b^	−0.7 (−1.4 to 0)	.06	0.52
6 mo	55	−1.0 (1.4)^b^	−1.8 (1.4)^b^	−0.9 (−1.6 to −0.1)	.02	0.57
Change in fasting blood glucose, mg/dL						
3 mo	55	−23.4 (45.1)^b^	−34.2 (59.5)^b^	−12.6 (−39.6 to 16.2)	.40	0.20
6 mo	55	−16.2 (45.1)	−41.4 (54.1)^b^	−25.2 (−50.5 to 0)	.10	0.51
**HbA_1c_<8% subgroup**^**a**^** (control group, 76 participants; intervention group, 73 participants)**						
Change in HbA_1c_, %						
3 mo	149	−0.1 (0.5)	−0.4 (0.6)^b^	−0.3 (−0.5 to −0.1)	<.001	0.54
6 mo	149	−0.1 (0.6)	−0.3 (0.7)^b^	−0.2 (−0.4 to 0)	.03	0.31
Change in fasting blood glucose, mg/dL						
3 mo	149	3.6 (21.6)	−12.6 (23.4)^b^	−14.4 (−21.6 to −7.2)	<.001	0.72
6 mo	149	1.8 (21.6)	−7.2 (32.4)^b^	−9.0 (−18.0 to 0)	.03	0.33

Abbreviation: HbA_1c_, Hemoglobin A_1c._

SI conversion factors: To convert HbA_1c_ to proportion of total hemoglobin, multiply by 0.01; glucose to millimoles per liter, multiply by 0.0555.

^a^ Results are presented for imputed data using the Markov chain Monte Carlo method.

^b^ Statistically significant change from baseline to postintervention at *P* < .05.

### Dietary Intake and Physical Activity

At 6 months, the app intervention led to reductions in total energy, carbohydrate, sugar, total fat, and saturated fat intake, along with an increase in physical activity. There were statistically significant between-group differences for all (eg, mean difference in physical activity: 62.4 min/wk; 95% CI, 16.1-108.6 min/wk; *P* = .009) (Table 2).

### Diabetes Medications

In the post hoc analysis of the subgroup taking diabetes medications, a significantly greater proportion of participants in the intervention group were found to have their diabetes medications reduced compared with participants in the control group (17 participants [23.3%] vs 4 participants [5.4%]), corresponding to a relative risk (RR) of 3.5 for reduction in diabetes medications (95% CI, 1.2-10.7; *P* = .03) (eTable 3 in Supplement 1). Conversely, a greater proportion of participants in the control group had their medications increased compared with the intervention group (18 [24.3%] vs 5 [6.8%]; RR, 0.3; 95% CI, 0.1-0.9; *P* = .04). These overall changes in medications led to a reduction in annual costs of diabetes medications in the intervention group, but an increase in the control group, with statistical significance between-group differences (mean difference in cost at 6 mo, −$145.30; 95% CI, −$252.40 to −$38.30; *P* = .01) (Table 2).

### Adverse Events

Mild hypoglycemia was reported by 3 participants in the intervention group, with none requiring hospitalization. No serious adverse events were reported.

### App Usage

App usage was defined as days when participants used at least 1 app feature. Overall, 61 of 99 (62%) of the intervention participants used the app at least 75% of the days during the 6-month intervention. Median (interquartile range [IQR]) days of app utilization for the 1 to 3–month, 4 to 6–month, and 6-month periods were 87 (69-91), 76 (36-90), and 161 (104-180) days, respectively. Median (IQR) days when participants communicated with a dietitian via the app were 16 (10-25), 6 (1-12), and 23 (11-36) during 1 to 3–month, 4 to 6–month, and 6-month periods, respectively.

## Discussion

The D’LITE study demonstrated that a culturally contextualized smartphone-based lifestyle intervention is capable of achieving meaningful weight reductions among Asian adults with type 2 diabetes and overweight or obesity who are not receiving insulin. In addition, the app intervention led to significant glycemic improvements, particularly among individuals with suboptimal diabetes control, while reducing the dosages and costs of diabetes medications. As there was a higher proportion of men than women, the weight improvement seen may be attenuated compared with other studies^37^ which tend to include predominantly women. There is also a 2015 systematic review^38^ demonstrating that men tend to lose more weight.

Weight loss from the app intervention in this study was similar to that achieved with previous face-to-face lifestyle interventions in individuals with diabetes, despite reduced face-to-face interactions.^5,37,39^ Typically, lifestyle intervention studies involve 3 to 12 visits in the initial 3 to 6 months, with a total duration of 2 to 16 hours.^5,40^ Because the ease of communication in the app interface allows an increased frequency of touch points between health care professionals and users to facilitate the provision of timely advice at the point of decision-making, it is able to mitigate the potential effects of reduced face-to-face interactions, leading to comparable outcomes.

With the present weight loss results being sustained from 3 to 6 months in spite of reduced interactions between health care professionals and users, it is plausible that the inclusion of self-management app features such as self-monitoring, automated feedback, prompts, and educational videos facilitated self-empowerment to reduce the reliance on health care professionals over time, which may potentially translate to manpower cost savings.

In parallel with the 4.3% weight loss achieved by the intervention group at 6 months, which meets the minimal 3% recommended for insulin resistance improvements,^8,9^ we found improvements in HbA_1c_ and FBG of similar magnitude to those achieved through face-to-face lifestyle interventions.^5,39,41,42^ Importantly, the present intervention produced a more pronounced HbA_1c_ improvement among those with suboptimal glycemic control, an effect greater than that achieved with most oral glucose-lowering agents.^43^ This would have translated to significant long-term protection against microvascular and macrovascular complications,^44,45^ suggesting that greater effort should be put in place to optimize lifestyle rather than adding on medications.

We also found that HbA_1c_ reduction was achieved despite reductions in diabetes medications in the intervention group, agreeing with results from medical nutrition therapy interventions in previous studies.^46,47,48,49^ Individuals with diabetes incur a notably higher health care expenditure compared with individuals without diabetes.^50^ In tandem with the reduction in diabetes medications, the app-led lifestyle intervention has the potential to reduce pill burdens, translating to lower medication costs while potentially lowering exposure to medication-associated adverse events.

Previous RCTs on smartphone-based interventions among Asian adults with type 2 diabetes have shown significantly greater improvements in HbA_1c_ levels in intervention groups compared with control groups following intervention periods of 3 to 12 months, but not for weight.^24,25,26,27,28^ In the present study, the phone app and in-app coaching helped participants achieve comparable glycemic improvements while concomitantly improving weight. The inclusion of features within the app, such as a weight tracking function; automated evaluation of calorie intake; alerts on foods logged that are high in fat, sugar, and sodium; and provision of healthier food alternatives may have accounted for the differences observed.

We also observed that in the intervention group, weight and glycemic improvements were in line with the adoption of a healthier lifestyle.^51^ There were greater reductions in intake of total energy, specifically from carbohydrate, sugar, fat and saturated fat in the app group, and a concomitant increase in physical activity.

One of our study’s strengths lay in it being a stratified RCT, which ensured similar baseline characteristics between groups. The use of intention-to-treat analysis accounted for all patients enrolled in the study, thus minimizing type I error and allowing for generalizability. The multicenter approach for recruitment enabled a more representative sample of the population. The attrition rate of 5% is relatively low compared with dietetics intervention studies conducted mainly in outpatient clinics^15^ and might be attributable to the ease and convenience of an app-based intervention, as it does away with both traveling time or the need to plan around a scheduled session. In addition, the use of multiple imputation method to account for missing values in this study reduced bias due to selective attrition.

### Limitations

This study has several limitations. Because of the varying effects of different antidepressants on weight, participants with depression were excluded from the study, and hence the sample might not be fully representative of the target population. We had selected smartphone users who were literate in English, thus potentially introducing selection bias, and which may have limited the generalizability of the study. Nonetheless, smartphone ownership and usage is on the rise globally, including in Singapore, where 92% of Singapore residents reported recent smartphone usage.^52,53^ Lifestyle intervention using an app with instant feedback and remote dietitian support could potentially serve the wider population in near future. This is especially significant in the face of the COVID-19 pandemic, particularly for communities where medical services may not be easily accessible.

Results for the outcome on physical activity have to be interpreted with caution because this study used self-reporting and lacked a validated measure. We also did not compare the relative contributions of different app components with weight and glycemic improvements, which may have helped to map the specific app features to outcomes. Furthermore, the long-term lifestyle and behavioral changes after the intervention period are still ongoing at the time of this writing and will be presented in a separate article.

## Conclusions

This study found that a culturally contextualized smartphone-based lifestyle intervention using a phone app with in-app coaching was capable of achieving significant improvements in weight and multiple metabolic profiles within 3 months of intervention that were sustainable at 6 months of intervention. Participants in the app intervention had also adopted healthier dietary and exercise habits. Thus, apps may offer a platform for the delivery of lifestyle interventions to benefit individuals with diabetes. Future research can investigate the specific combination of app features that are most likely to achieve successful outcomes, as well as the effectiveness of such apps in other Asian populations.

## Notes

Supplement 1. **eAppendix.** nBuddy Diabetes Mobile App **eFigure 1.** Behavioral Treatment Strategies Incorporated into nBuddy Diabetes App to Optimize Blood Glucose Level and Weight Loss **eFigure 2.** Screenshots of the nBuddy Diabetes Mobile App eReferences **eTable 1.** Primary and Secondary Outcomes at 3 and 6 Months After Enrollment Using Complete Case Analysis **eTable 2.** Changes in HbA1c and Fasting Blood Glucose at 3 and 6 Months of Intervention for Subgroups HbA1c ≥ 8% and HbA1c < 8% Using Complete Case Analysis **eTable 3.** Changes in the Dosage of Diabetes Medications at 6 Months After Enrollment Using Complete Case and Multiple Imputation Analysis

Supplement 2. Trial Protocol

Supplement 3. Data Sharing Statement
