# Supplementary material for: Effect of a Smartphone App on Weight Change and Metabolic Outcomes in Asian Adults With Type 2 Diabetes: A Randomized Clinical Trial
Source: JAMA Netw Open. 2021 Jun 3;4(6):e2112417. doi: 10.1001/jamanetworkopen.2021.12417 (PMC8176331; doi:10.1001/jamanetworkopen.2021.12417)
Supplement: Supplement 1. — eAppendix. nBuddy Diabetes Mobile App eFigure 1. Behavioral Treatment Strategies Incorporated into nBuddy Diabetes App to Optimize Blood Glucose Level and Weight Loss eFigure 2. Screenshots of the nBuddy Diabetes Mobile App eReferences eTable 1. Primary and Secondary Outcomes at 3 and 6 Months After Enrollment Using Complete Case Analysis eTable 2. Changes in HbA1c and Fasting Blood Glucose at 3 and 6 Months of Intervention for Subgroups HbA1c ≥ 8% and HbA1c < 8% Using Complete Case Analysis eTable 3. Changes in the Dosage of Diabetes Medications at 6 Months After Enrollment Using Complete Case and Multiple Imputation Analysis [file jamanetwopen-e2112417-s001.pdf]

Supplemental Online Content

Lim SL, Ong KW, Johal J, et al. Effect of a smartphone app on weight change and metabolic outcomes in Asian adults with type 2 diabetes: a randomized clinical trial. *JAMA Netw Open*. 2021;4(6):e2112417. doi:10.1001/jamanetworkopen.2021.12417

eAppendix. nBuddy Diabetes Mobile App
eFigure 1. Behavioral Treatment Strategies Incorporated Into nBuddy Diabetes App to Optimize Blood Glucose Level and Weight Loss
eFigure 2. Screenshots of the nBuddy Diabetes Mobile App
eReferences
eTable 1. Primary and Secondary Outcomes at 3 and 6 Months After Enrollment Using Complete Case Analysis
eTable 2. Changes in HbA1c and Fasting Blood Glucose at 3 and 6 Months of Intervention for Subgroups HbA1c ≥ 8% and HbA1c < 8% Using Complete Case Analysis
eTable 3. Changes in the Dosage of Diabetes Medications at 6 Months After Enrollment Using Complete Case and Multiple Imputation Analysis

This supplemental material has been provided by the authors to give readers additional information about their work.

© 2021 Lim SL et al. *JAMA Network Open.*

eAppendix. nBuddy Diabetes mobile app

The nBuddy Diabetes was conceptualized by the principal investigator (SLL) and developed by HeartVoice. It was developed using the Obesity-Related Behavioral Intervention Trials (ORBIT) Model for behavioral intervention as a framework for translating behavioral science into treatments, as it is a flexible and robust process, to design, conduct and evaluate mobile-app based behavioral interventions.[1] The app was conceptualized based on the construct of theory which is depicted in eFigure 1. The app is available to the public in the app stores, with basic features accessible for free. Payment can be made to access additional features such as daily tips, videos, and nutritionist support.

The following set of features in nBuddy Diabetes is an integration of evidence-based behavioral modification strategies to promote weight loss/maintenance:

- Self-monitoring of food/calorie intake is enabled using a diary logging system, coupled with individualized caloric and carbohydrate goals based on user's age, gender and physical activity levels.[2]
- An automated evaluation of calorie, carbohydrate and sugar intake, with instantaneous feedback provided if calorie and/or carbohydrate limits have been exceeded for the meal or day.
- Self-monitoring of physical activity is facilitated by syncing with users' mobile devices' in-built pedometer.[3] Step goal increases automatically from 3000 to 10,000 by the third week of usage. Users have the option of logging in a range of physical activities manually if exercises were done in absence of their mobile devices.
- An automated response system evaluates suitability of food choices and provides instantaneous feedback to generate a list of healthier, diabetes-friendly and culturally appropriate food alternatives via an algorithm.[4]

© 2021 Lim SL et al. *JAMA Network Open.*

- Self-tracking of weight loss progression is enabled via the weight logging function.[5]
- Self-tracking of blood glucose level can be carried out using the glucose logging function.[6] Participants are recommended to monitor their fasting and 2-hour post-prandial blood glucose 2 days weekly in the first 3 months.
- A peer support chat channel allows family members and peers to support users to bolster motivation.[3]
- A video-viewing function delivers short educational clips to participants weekly.[7]
- Provision of daily, weekly and monthly graph reports on weight, calorie intake, carbohydrate intake, steps count and blood glucose facilitates the tracking of progress.[2]
- Scripted daily tips and timed reminders prompt users to log in their food, weight and blood glucose regularly.[8]
- A dashboard allows user progress (i.e. food intake and physical activity) and outcomes (i.e. weight and blood glucose) to be monitored by dietitians, to enable the provision of real-time feedback and encouragement.[9,10]

© 2021 Lim SL et al. *JAMA Network Open.*

eFigure 1. Behavioural treatment strategies incorporated into nBuddy Diabetes App to optimise blood glucose level and weight loss

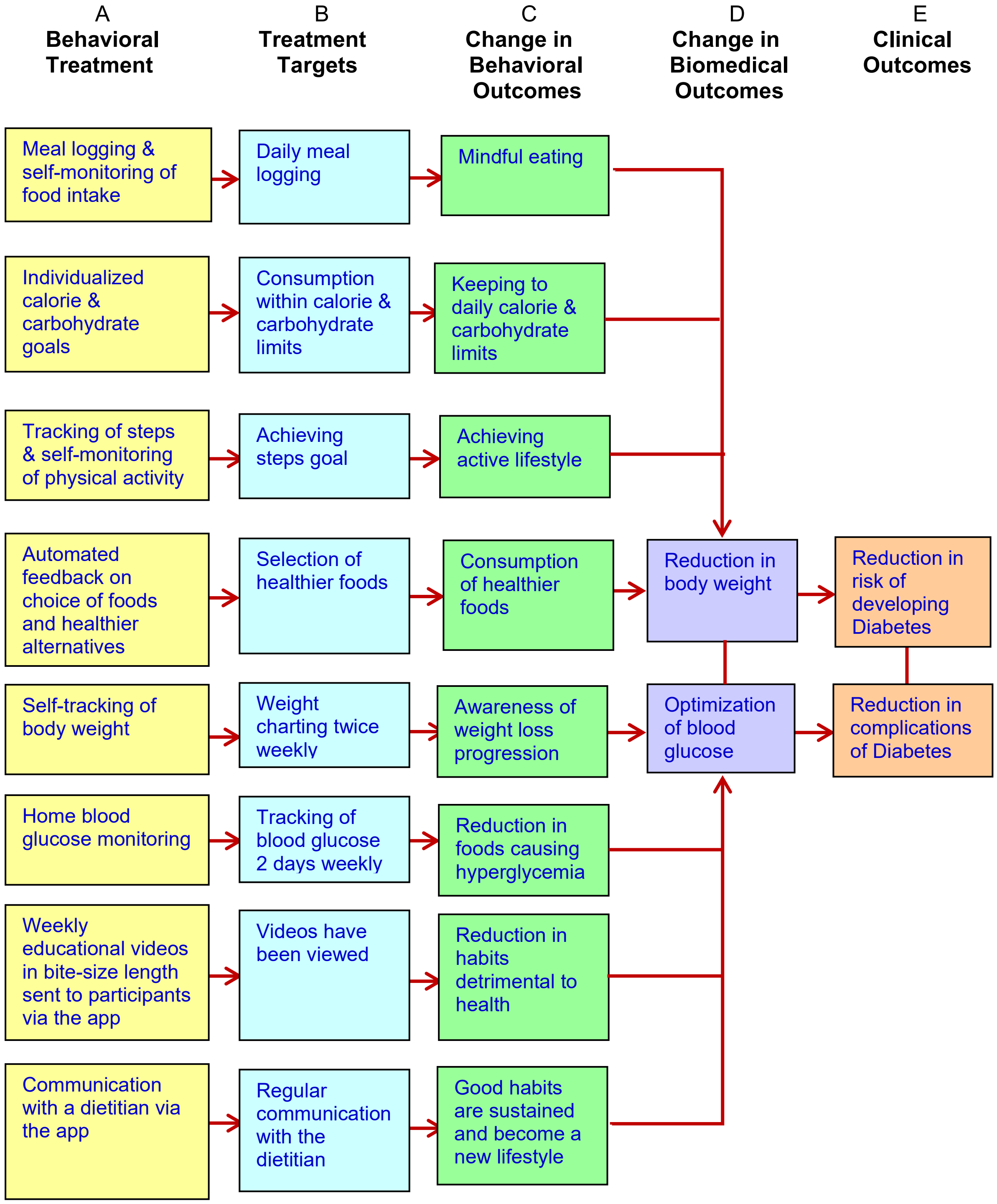

© 2021 Lim SL et al. *JAMA Network Open.*

eFigure 2: Screenshots of the nBuddy Diabetes Mobile App

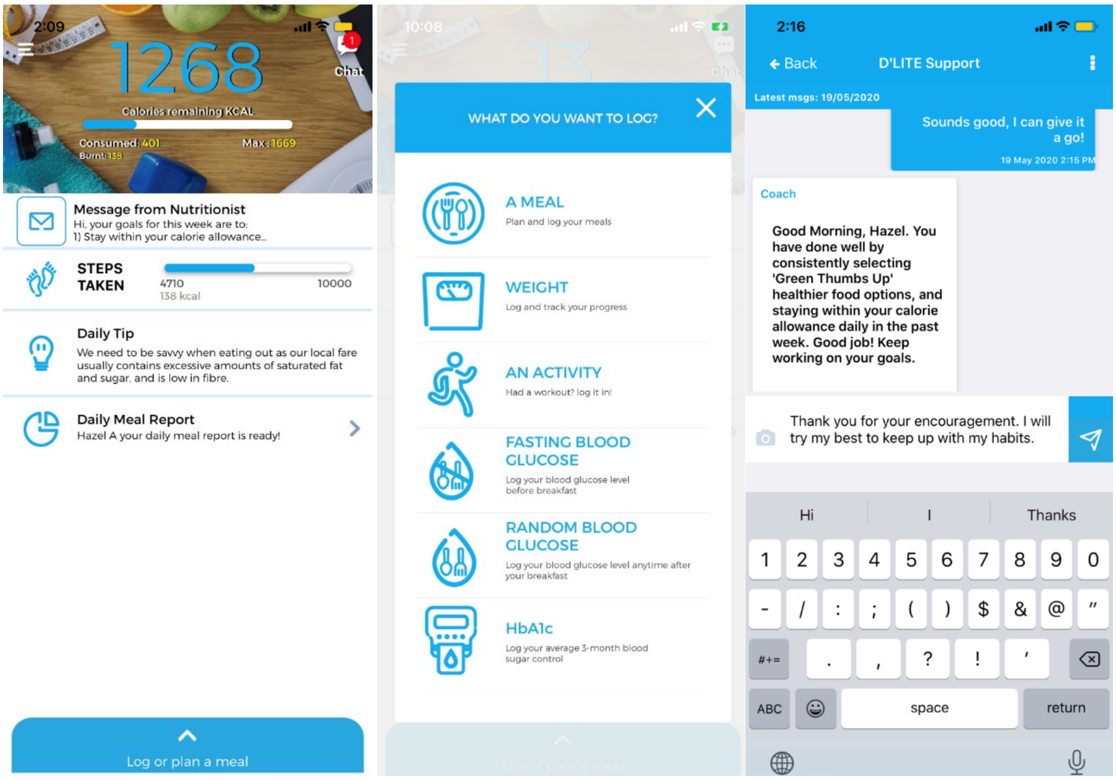

© 2021 Lim SL et al. *JAMA Network Open.*

eReferences

1. Czajkowski SM, Powell LH, Adler N, et al. From ideas to efficacy: The ORBIT model for developing behavioral treatments for chronic diseases. *Health Psychology.* 2015;34(10):971.
2. Burke LE, Wang J, Sevick MA. Self-monitoring in weight loss: a systematic review of the literature. *Journal of the American Dietetic Association.* 2011;111(1):92-102.
3. Mohr D, Cuijpers P, Lehman K. Supportive accountability: a model for providing human support to enhance adherence to eHealth interventions. *Journal of medical Internet research.* 2011;13(1):e30.
4. Chance Z, Gorlin M, Dhar R. Why choosing healthy foods is hard, and how to help: presenting the 4Ps framework for behavior change. *Cust Needs Solut.* 2014;1(4):253-262.
5. Rao G, Burke LE, Spring BJ, et al. New and emerging weight management strategies for busy ambulatory settings: a scientific statement from the American Heart Association endorsed by the Society of Behavioral Medicine. *Circulation.* 2011;124(10):1182.
6. Cui M, Wu X, Mao J, Wang X, Nie M. T2DM self-management via smartphone applications: a systematic review and meta-analysis. *PloS One.* 2016;11(11).
7. Tuong W, Larsen ER, Armstrong AW. Videos to influence: a systematic review of effectiveness of video-based education in modifying health behaviors. *Journal of behavioral medicine.* 2014;37(2):218-233.
8. Klasnja P, Pratt W. Healthcare in the pocket: mapping the space of mobile-phone health interventions. *J Biomed Inform.* 2012;45(1):184-198.
9. Butryn ML, Webb V, Wadden TA. Behavioral treatment of obesity. *Psychiatric Clinics.* 2011;34(4):841-859.
10. Mitchell LJ, Ball LE, Ross LJ, Barnes KA, Williams LT. Effectiveness of dietetic consultations in primary health care: a systematic review of randomized controlled trials. *J Acad Nutr Diet.* 2017;117(12):1941-1962.

© 2021 Lim SL et al. *JAMA Network Open.*

eTable 1. Primary and Secondary Outcomes at 3 and 6 months after Enrollment using Complete Case Analysis

Outcomes	*n*	Mean Change from Baseline: Control (*n* = 105)	Mean Change from Baseline: Intervention (*n* = 99)	Between-group differences: Mean difference (95% CI)	*P* value	*Cohen d*
Δ Weight, kg						
3 months	195	-0.7 (2.2)*	-3.0 (3.5)*	-2.4 (-3.2 to -1.5)	<.001	0.79
6 months	193	-1.0 (2.6)*	-3.5 (4.5)*	-2.5 (-3.5 to -1.4)	<.001	0.68
Δ Weight, %						
3 months	195	-0.8 (2.6)	-3.6 (4.0)	-2.8 (-3.8 to -1.8)	<.001	0.83
6 months	193	-1.2 (3.1)	-4.2 (5.2)	-3.0 (-4.2 to -1.8)	<.001	0.70
Δ BMI, kg/m2						
3 months	195	-0.2 (0.8)*	-1.1 (1.2)*	-0.9 (-1.2 to -0.6)	<.001	0.88
6 months	193	-0.4 (0.9)*	-1.3 (1.6)*	-0.9 (-1.3 to -0.5)	<.001	0.69
Δ HbA_{1c}, %						
3 months	195	-0.3 (0.9)*	-0.7 (1.0)*	-0.4 (-0.7 to -0.2)	.001	0.42
6 months	192	-0.3 (0.9)*	-0.7 (1.1)*	-0.4 (-0.7 to -0.2)	.003	0.40
ΔFasting Blood Glucose, mg/dL						
3 months	193	-3.6 (30.6)	-16.2 (36.0)*	-12.6 (-23.4 to -3.6)	.005	0.38
6 months	187	-1.8 (27.0)	-16.2 (39.6)*	- 14.4 (-25.2 to -5.4)	.002	0.42
Δ Systolic blood pressure (mmHg)		*n* = 72	*n* = 67			
3 months	132	-1.4 (13.8)	-6.1 (12.6)*	-4.7 (-9.3 to -0.2)	.04	0.36
6 months	131	-4.4 (13.2)*	-7.9 (14.2)*	-3.5 (-8.2 to 1.3)	.14	0.26
Δ Diastolic blood pressure (mmHg)						
3 months	132	-1.6 (10.6)	-4.1 (8.8)*	-2.6 (-5.9 to 0.8)	.13	0.26
6 months	131	-2.5 (7.9)*	-5.4 (10.9)*	-2.9 (-6.2 to 0.4)	.08	0.30
Δ Annual cost of diabetes medications, S$		*n* = 74	*n* = 73			
3 months	141	10.4 (102.3)	-62.1 (266.7)	-72.5 (-139.6 to -5.4)	.03	0.36
6 months	136	79.9 (284.4)*	-72.9 (329.8)	-152.8 (-257.1 to -48.4)	.004	0.50
ΔTotal cholesterol mmol/L		*n* = 71	*n* = 72			
3 months	135	-0.08 (0.84)	-0.33 (0.62)*	-0.26 (-0.51 to 0)	.04	0.34
6 months	135	-0.16 (1.07)	-0.25 (0.85)*	-0.07(-0.39 to 0.26)	.68	0.09
ΔLDL cholesterol, mmol/L						
3 months	134	-0.03 (0.67)	-0.19 (0.56)*	-0.16 (-0.37 to 0.05)	.13	0.26
6 months	134	-0.09 (0.85)	-0.17 (0.75)	-0.07 (-0.34 to 0.20)	.62	0.10
ΔHDL cholesterol mmol/L						
3 months	135	0.02 (0.16)	0.03 (0.26)	0.01 (-0.06 to 0.09)	.76	0.05
6 months	135	0.02 (0.16)	0.03 (0.15)	0.01 (-0.05 to 0.06)	.79	0.06

© 2021 Lim SL et al. *JAMA Network Open.*

ΔTriglyceride, mmol/L						
3 months	135	-0.24 (1.14)	-0.40 (0.62)*	-0.16 (-0.47 to 0.15)	.30	0.17
6 months	135	-0.33 (1.14)*	-0.25 (0.72)*	0.07 (-0.25 to 0.40)	.66	0.08
Δ Creatinine, umol/L						
3 months	194	0.1 (8.8)	-0.2 (7.6)	-0.2(-2.6- 2.1)	.84	0.04
6 months	190	1.7 (9.1)	0.9 (8.6)	-0.8(-3.3- 1.7)	.53	0.09
Δ Calorie, kcal						
3 months	193	-204.3 (571.2)*	-587.7 (551.2)*	-383.4 (-543.0 to -223.8)	<.001	0.68
6 months	189	-223.5 (443.1)*	-572.0 (514.5)*	-348.5 (-486.0 to -211.1)	<.001	0.73
ΔCarbohydrate, g						
3 months	193	-25.0 (64.1)*	-67.1 (70.0)*	-42.1 (-61.1 to -23.1)	<.001	0.63
6 months	189	-25.7 (60.5)*	-66.6 (62.6)*	-41.0 (-58.7 to -23.3)	<.001	0.66
Δ Sugar, g						
3 months	193	-9.7 (33.6)*	-22.0 (31.7)*	-12.3 (-21.6 to -3.0)	.01	0.38
6 months	189	-9.3 (30.8)*	-23.1 (27.4)*	-13.8 (-22.2 to -5.4)	<.001	0.47
Δ Protein, g						
3 months	193	-4.8 (29.7)	-16.0 (28.4)*	-11.2 (-19.4 to -2.9)	.008	0.39
6 months	189	-7.7 (26.7)*	-14.5 (24.8)*	-6.8 (-14.3 to 0.6)	.07	0.26
Δ Total fat, g						
3 months	193	-6.8 (30.8)*	-29.3 (32.5)*	-22.6 (-31.5 to -13.6)	<.001	0.71
6 months	189	-9.0 (21.7)*	-27.4 (31.9)*	-18.4 (-26.2 to -10.7)	<.001	0.67
Δ Saturated fat, g						
3 months	193	-3.9 (14.1)*	-11.9 (13.7)*	-8.1 (-12.0 to -4.2)	<.001	0.58
6 months	189	-3.9 (10.7)*	-12.3 (14.0)*	-8.4 (-12.0 to -4.9)	<.001	0.67
Δ Fiber, g						
3 months	193	-0.8 (8.2)	-3.7 (7.2)*	-2.9 (-5.1 to -0.7)	.01	0.38
6 months	189	-2.0 (6.6)*	-2.9 (7.3)*	-0.9 (-2.9 to 1.1)	.39	0.13
ΔPhysical activity, minutes per week						
3 months	193	13.6 (100.3)	67.7 (173.0)*	54.0 (14.2 to 93.8)	.008	0.38
6 months	188	5.7 (119.0)	67.0 (171.5)*	61.3 (19.1 to 103.4)	.005	0.42

Data are mean ± SD
* Significant within-group changes p values after Benjamini-Hochberg correction with false discovery rate at 0.20 and n=80.

Abbreviations: BMI, Body Mass Index (calculated as weight in kilograms divided by height in meters squared); HbA_{1c}, Hemoglobin A_{1c}; LDL, low-density lipoprotein; HDL, high-density lipoprotein.

SI conversion factors: To convert HbA_{1c} to proportion of total hemoglobin, multiply by 0.01; glucose to mmol/L, multiply by 0.0555; total, HDL and LDL cholesterol to mmol/L, multiply by 0.0259; triglycerides to mmol/L, multiply by 0.0113

© 2021 Lim SL et al. *JAMA Network Open.*

eTable 2: Changes in HbA_{1c} and Fasting Blood Glucose at 3 and 6 Months of Intervention for Subgroups $HbA_{1c} \geqslant 8\%$ and $HbA_{1c} < 8\%$ using Complete Case Analysis

Outcome Variables	*n*	Change from Baseline		Between-group differences		
		Control (*n* = 29)	Intervention (*n* = 26)	Mean difference (95% CI)	*P* value	*Cohen d*
**Subgroup $HbA_{1c} \geqslant 8\%$**						
Δ HbA_{1c}, %						
3 months	52	-1.0 (1.2)*	-1.8 (1.4)*	-0.8 (-1.5 to -0.1)	.03	0.61
6 months	50	-0.9 (1.3)*	-1.8 (1.4)*	-1.0 (-1.7 to -0.2)	.01	0.67
ΔFasting Blood Glucose, mg/dL						
3 months	51	-21.6 (45.0)*	-28.8 (57.6)*	-7.2 (-36.0 to 21.6)	.60	0.14
6 months	46	-12.6 (36.0)	-41.4 (50.4)*	-28.8 (-54.0 to -1.8)	.03	0.66
**Subgroup $HbA_{1c} < 8\%$**						
Δ HbA_{1c}, %						
3 months	144	0 (0.5)	-0.4 (0.5)*	-0.3 (-0.5 to -0.2)	<.001	0.80
6 months	142	-0.1 (0.6)	-0.3 (0.7)*	-0.2 (-0.5 to 0)	.02	0.31
Δ Fasting Blood Glucose, mg/dL						
3 months	142	3.6 (19.8)	-12.6 (21.6)*	-16.2 (-23.4 to -9.0)	<.001	0.78
6 months	141	1.8 (21.6)	-9.0 (32.4)*	-10.8 (-19.8 to -1.8)	.01	0.39

Abbreviation: HbA_{1c}, Hemoglobin A_{1c}
SI conversion factors: To convert HbA_{1c} to proportion of total hemoglobin, multiply by 0.01; glucose to mmol/L, multiply by 0.0555
*Statistically significant change from baseline to post intervention at $P < .05$

© 2021 Lim SL et al. *JAMA Network Open.*

eTable 3: Changes in the Dosage of Diabetes Medications at 6 months after Enrollment using Complete Case and Multiple Imputation Analysis

	Complete Case Analysis (n=136)			Multiple Imputation Analysis (n=147)[a]		
	Control n (%)	Intervention n (%)	RR (95% CI) *P* value	Control n (%)	Intervention n (%)	R (95% CI) *P* value
No change	51 (74.0)	50 (74.6)	N.A.	52 (70.3)	51 (69.9)	N.A.
Reduction in Diabetes Medication	3 (4.3)	15 (22.4)	4.2 (1.2-14.5) .026	4 (5.4)	17 (23.3)	3.5 (1.2-10.7) .028
Increase in Diabetes Medication	15 (21.7)	2 (3.0)	0.17 (0.04-0.75) .020	18 (24.3)	5 (6.8)	0.34 (0.13-0.94) .044

RR: Relative Risks are Intervention vs Control
[a]Multiple imputation method using Markov Chain Monte-Carlo approach was utilized
N.A.: Not applicable

© 2021 Lim SL et al. *JAMA Network Open.*
