# Supplementary material for: Effect of a Smartphone App on Weight Change and Metabolic Outcomes in Asian Adults With Type 2 Diabetes: A Randomized Clinical Trial
Source: JAMA Netw Open. 2021 Jun 3;4(6):e2112417. doi: 10.1001/jamanetworkopen.2021.12417 (PMC8176331; doi:10.1001/jamanetworkopen.2021.12417)
Supplement: Supplement 2. — Trial Protocol [file jamanetwopen-e2112417-s002.pdf]

Supplement 2

Diabetes Lifestyle Intervention using Technology Empowerment (D'LITE)

Study Protocol (for Diabetes Cohort)

Background

The increasing prevalence of type 2 diabetes mellitus and the consequent burden on healthcare resources is a major concern in Singapore and many countries around the world. In 2010, 25.6% of Singapore's adult population aged 18-69 years old have diabetes or pre-diabetes.[1] It was estimated that $1 billion was spent on diabetes in 2010 and this will soar to $2.5 billion in 2050.[2]

The rapidly aging population in Singapore will only aggravate the situation. The burden of diabetes lies not only in bringing physiological abnormalities (such as high blood sugar and excess weight) back to normal, but more so in preventing the associated complications, which may lead to severe morbidities, escalating healthcare costs and premature deaths. Untreated and uncontrolled diabetes leads to coronary heart disease, renal failure, blindness, limb amputations and many other end-organ complications.[3] Current treatment of diabetes mellitus is unfortunately reactive and often started late in the disease process. By the time patients present with overt symptoms, the condition has already wreaked havoc on vital organs and blood vessels in the body, and healthcare professionals are often reactively "fighting fires". It is unfortunate that many patients only get to see the dietitians for the first time when they are faced with the complications of diabetes. Management of diabetes needs to move upstream, to the prevention of disease in the first place, and optimal control of disease once it has been diagnosed to prevent further complications.

Lifestyle intervention programs commonly incorporate diet, exercise, behaviour modifications, consultations with a dietitian or exercise therapist (or both), and a customized diet or exercise plan (or both). It has been shown that success of the treatment is associated with the intensity of lifestyle measures and can lead to greater weight loss in those who are overweight or obese, and a reduction in new-onset diabetes.[4-8] However, studies showing positive results were mostly conducted in controlled environments which may or may not be transferable to the local (Singapore) context.

Although lifestyle interventions such as diet and exercise programs are considered fundamental in the prevention and management oftype 2 diabetes,[9] current methods of delivering them are not scalable to the at-risk population as they are resource-intensive, costly, time-consuming, have high default rate and limited reach.

In 2014, the mobile population penetration rate in Singapore was 148%, as it was not uncommon for individuals to own two or more mobile phones, and additionally 87% of households had access to broadband.[10,11] These numbers will only increase with time. Increased connectivity, advancing technology and innovative care delivery models open up many opportunities to improve the way we prevent and manage chronic diseases.

Behavior plays a key role in the prevention and management of type 2 diabetes. An estimated 40% of premature deaths are attributable to preventable behavioral factors such as unhealthy dietary intake and sedentary lifestyle.[12-15] In recent years, basic behavioral and social sciences research are looking promising in terms of addressing behaviorally-influenced health problems, particularly chronic diseases. There is an early and growing body of knowledge on the use of mobile and other technologies for behavior change in health and disease.[16] Equipped with increasingly sophisticated sensing technology and powerful processors, smartphones can both unobtrusively measure behavior and be an ideal platform for delivering feedback and behavioral therapy.[17,18]

Modern technology-enabled interventions such as health-related mobile apps have been increasingly used in facilitating weight loss. However, this technology-enabled modality of treatment is currently fragmented with its weakest link being the diet aspect where compliance is poor due to it being user-unfriendly, not targeted in its advice and not being able to provide individualized diet according to culture and pre-existing medical conditions.[19] A few studies using mobile technologies to facilitate weight loss have been done overseas and mostly in Caucasian populations, but no such studies have been carried out on type 2 diabetes patients. A study using this modality of intervention and conducted in the local context will provide new knowledge on the feasibility and effectiveness of mobile technologies to facilitate lifestyle

interventions in people with diabetes and provide guidance for the application of this modality of treatment to other chronic diseases in future.

We propose to use a smartphone app and remote intervention programs to deliver lifestyle interventions to individuals with diabetes, with the appropriate targeted and individualized support, led and supervised remotely by dietitians. We will use a user-friendly smartphone app to facilitate lifestyle changes (diet and activity) with elements of behavioral modifications embedded, -, including remote coaching. The proposed study will design, implement and evaluate behavioral and lifestyle interventions, using combined smartphone mobile app and remote dietetic coaching program, specifically for effective lifestyle management of diabetes in Singapore.

The study hypothesis is that a locally-developed and targeted mobile app, combined with remote coaching, will be able to effect positive changes in health-related behaviors and lifestyle choices, manage diabetes, and ultimately improve health outcomes and save healthcare costs.

Objective

The objective of the Diabetes Lifestyle Intervention using Technology Empowerment (D'LITE) study is to compare the effectiveness of a weight loss lifestyle intervention, delivered via the nBuddy Diabetes app and in-app coaching by dietitians, with usual care, on body weight and metabolic profiles among overweight or obese non-insulin-treated Asians with type 2 diabetes.

Study Design

The D'LITE study is a parallel multicenter 2-arm randomized clinical trial (RCT). Participants will be from health screening facilities, government polyclinics, general practitioner clinics and hospital outpatient clinics in Singapore.

Inclusion criteria:

- Adults between 21-75 years old
- Diagnosed with type 2 diabetes
- BMI of 23 kg/m^2 or higher

- Own a smartphone with data plan
- Literate in English
- Give consent to the study

Exclusion criteria:

- Known severe cognitive or psychological disabilities
- Heart failure
- Stage 4 and above kidney disease
- Untreated hypothyroidism
- Depression
- Type 1 diabetes
- Insulin-treated type 2 diabetes
- Pregnancy
- Non-compliant to prescribed diabetes medications
- Untreated anemia, thalassemia or other blood disorder
- Not keen to participate

Randomization

Eligible participants were randomized to either control or intervention group in a 1:1 allocation ratio via block randomization stratified by gender, BMI (<27.5 or ≥ 27.5 kg/m^2), and age (<50 years or ≥ 50 years), which was changed from previous <40 years or ≥ 40 years 2 months post-recruitment due to a noticeably larger number of older participants. Participants were allocated to either group by drawing personally from sealed stratified opaque envelopes, each containing an equal proportion of intervention and control group assignments. To ensure high quality envelope concealment, a third party personnel not involved in the study prepared the envelopes in advance using matched block method.

Intervention

All control and intervention participants received a single 45 to 60 minutes session of diet and physical activity advice, as per American Dietetic Association (ADA) guidelines, from a registered research dietitian in the clinic at baseline. All participants were issued a standardized digital weighing scale (Omron HN-289, Japan), and continued to receive standard diabetes care from their usual health care providers.

Participants assigned to the intervention group were required to use the nBuddy Diabetes app for 6 months to track weight twice weekly, diet and physical activity daily and to communicate regularly with the research dietitians via the app. To encourage self-blood glucose monitoring, the intervention group was also provided with a glucometer. Based on the participants' input, the dietitian will provide personalized diet and lifestyle coaching. They will be guided to achieve their individualized goals using in-built evidence based behavior modification tools via the app. These are implemented as daily tips, prompters and decision-support system to motivate them towards their weight goal and blood glucose control. Participants can also review their progress via the weight, calorie intake, physical activity and blood glucose charts in the app. Educational videos will be uploaded for participants weekly via the application to enhance their knowledge in diabetes management.

Outcomes

The primary outcome of this study is weight loss, measured by a standardized digital weighing scale at 3-month and 6-month post randomisation. Secondary outcomes were changes in body weight post 3 months, metabolic profiles (HbA1c, fasting blood glucose (FBG), blood pressure, total cholesterol, triglycerides, low-density and high-density lipoprotein levels), creatinine and dietary intake. These measurements will be re-measured at 1-year and 2-year to assess long term sustainability of the outcomes. Blood samples will be processed at accredited laboratories - National University Hospital Referral laboratories and National Healthcare Group Diagnostics, with technicians blinded to the group allocation.

Participants in both groups will be required to complete simple ethics-approved surveys at baseline, 3-month, 6-month, 1-year and 2-year. Questions include participants' experience in using mobile apps, study expectations, stage of change, previous diet advice, dietary patterns, physical activity, medication changes and healthcare costs. At year 1 and 2, participants will also be required to complete a lifestyle and behavior questionnaire to better understand their weight loss goals, self-efficacy, physical activity, sedentary hours, factors leading to their weight outcomes and eating behavior.

A 2-day food diary, reflecting one weekday and one weekend dietary patterns, will be administered at baseline and subsequent visits. Participants are given basic instructions to list out foods or drinks consumed and to estimate the portion sizes with standard household

measures. To assess the change in energy and other macronutrients intake from baseline, 2-day food diary will be analyzed using the nBuddy Diabetes app's nutrient analysis database at every time point, which incorporates Singapore Energy & Nutrient Composition of Food, Malaysian Food Composition and USDA food databases, along with nutritional information from food packaging, and nutrient analysis of recipes.

Sample Size

The sample size is calculated based on assumption of at least a moderate Cohen's effect size of 0.5 for the difference in weight loss between groups at 6 months post randomization. A minimum sample size of 85 participants per group would provide 90% power at 0.05 level of significance (two-sided). A total sample size of 190 participants (95 per group) was planned, factoring a 10% attrition rate.

Statistical Analysis

All analyses will be performed using SPSS for Windows software (SPSS Inc, Chicago, IL, USA). Descriptive statistics for normally distributed numerical variables will be presented as mean (standard deviation), otherwise median (interquartile range) will be presented. Categorical data will be expressed as frequencies and percentages. Parametric tests (and the appropriate non-parametric) will be used to compare numerical variables between the control and intervention groups upon normality & homogeneity assumptions checking. For categorical variables, the chi-square or Fisher Exact tests will be used. Between-group differences in the primary outcomes (weight changes) and secondary outcomes (changes in HbA1c, fasting blood glucose, blood lipids, creatinine levels, blood pressure, calorie, carbohydrate, sugar and other nutrients intake, and physical activity) will be investigated using the Generalized Linear Mixed Model analysis, adjusted for baseline measurements and relevant covariates, and accounting for clustering effect of recruitment sources. Post hoc analysis using Generalized Poisson Mixed Model will be conducted to determine the relative risks for diabetic medication reduction. Statistical significance is set at $p < 0.05$. In the quantitative study, regression models will be used to test for significant treatment effects after controlling for confounders.

Summary of Changes to the Randomized Controlled Trial (RCT) Study Design

1) Recruitment was extended beyond community health screening to include the primary care clinics (government polyclinics or general practitioner GP clinics) and hospital outpatient clinics in order to boost recruitment rates and reach out to the target population.

2) The inclusion and exclusion criteria were further revised to ensure that the target population is reached:
   - Apart from fasting blood glucose as the only diagnostic criteria, known type 2 diabetes diagnosis was also added as part of the inclusion criteria.
   - Exclusion criteria was strengthened to include type 2 diabetes on insulin, non-compliance to diabetes medications, untreated anemia, thalassemia or other blood disorder, non-corresponding HbA1c with other blood glucose measurements, as clinically assessed by study team.

3) Criteria for stratified randomization was revised for age group from ≥ 40 and < 40 years old to ≥ 50 and < 50 years old for more uniformity between number of participants within groups.

4) Self-administered questionnaires were amended and ethics-approved to ensure that necessary data is obtained. Additional 1-year and 2-year lifestyle and behavior questionnaires were added to further examine the factors leading to weight loss maintenance.

5) Instead of using ANCOVA, Generalized Linear Mixed Model analysis was employed to account for clustering effect of recruitment sources for each of the numerical primary and secondary outcomes. Post-hoc analysis using Generalized Poisson Mixed Model was also conducted to determine the relative risks for medication reduction.

6) The primary outcome of this study is weight loss at 6 months, measured in the clinic using a calibrated digital weighing scale.

7) Secondary outcomes were expanded to include:
   a. Weight loss at 3-month post randomization

b. Changes in physical activity for all participants
c. HbA1c and FBG changes for subgroup with HbA1c ≥8%
d. Changes in diabetes medication dosages for subgroup on diabetes medications

8) Multiple imputations method was used to derive missing data points, with 5 imputations performed for each missing value using the Markov Chain Monte-Carlo method.

9) Benjamini-Hochberg procedure was also performed to adjust for multiple comparisons.

References

1. Epidemiology & Disease Control Division MoH, Singapore. National Health Survey 2010. In: Singapore.
2. Png ME, Yoong J, Phan TP, Wee HL. Current and future economic burden of diabetes among working-age adults in Asia: conservative estimates for Singapore from 2010-2050. *BMC Public Health.* 2016;16(1):153.
3. Knowler WC, Barrett-Connor E, Fowler SE, et al. Reduction in the incidence of type 2 diabetes with lifestyle intervention or metformin. *The New England journal of medicine.* 2002;346(6):393-403.
4. Gregg EW, Chen H, Wagenknecht LE, et al. Association of an Intensive Lifestyle Intervention With Remission of Type 2 Diabetes. *Jama.* 2012;308(23):2489-2496.
5. Li R, Qu S, Zhang P, et al. Economic evaluation of combined diet and physical activity promotion programs to prevent type 2 diabetes among persons at increased risk: a systematic review for the Community Preventive Services Task Force. *Ann Intern Med.* 2015;163(6):452-460.
6. Ministry of Health S. Clinical Practice Guidelines for Diabetes Mellitus. In: Singapore: Ministry of Health, Singapore; 2014.
7. Carter MC, Burley VJ, Nykjaer C, Cade JE. Adherence to a smartphone application for weight loss compared to website and paper diary: pilot randomized controlled trial. *J Med Internet Res.* 2013;15(4):e32.
8. Huang MA, Greenson JK, Chao C, et al. One-year intense nutritional counseling results in histological improvement in patients with non-alcoholic steatohepatitis: a pilot study. *Am J Gastroenterol.* 2005;100(5):1072-1081.
9. Balk EM, Earley A, Raman G, Avendano EA, Pittas AG, Remington PL. Combined Diet and Physical Activity Promotion Programs to Prevent Type 2 Diabetes Among Persons at Increased Risk: A Systematic Review for the Community Preventive Services Task Force. *Ann Intern Med.* 2015;163(6):437-451.
10. Authority IMD. Statistics on Telecom Services for 2015 (Jan - Jun). 2015. https://www.imda.gov.sg/infocomm-media-landscape/research-and-statistics/telecommunications/statistics-on-telecom-services/statistic-on-telecom-service-for-2015-jan.
11. Singapore IDAo. *Annual Survey on Infocomm Usage in Households and by Individuals for 2014.*
12. Mokdad AH, Marks JS, Stroup DF, Gerberding JL. Actual causes of death in the United States, 2000. *Jama.* 2004;291(10):1238-1245.

13. Schroeder SA. We Can Do Better — Improving the Health of the American People. *New England Journal of Medicine.* 2007;357(12):1221-1228.
14. Chakravarty EF, Hubert HB, Krishnan E, Bruce BB, Lingala VB, Fries JF. Lifestyle Risk Factors Predict Disability and Death in Healthy Aging Adults. *The American Journal of Medicine.* 2012;125(2):190-197.
15. Yoon P, Bastian B, Anderson RN, et al. *Potentially Preventable Deaths Among the Five Leading Causes of Death — United States, 2010 and 2014.* USA: Centers for Disease Control and Prevention; 2 May 2014 2014.
16. Levine DM, Savarimuthu S, Squires A, Nicholson J, Jay M. Technology-assisted weight loss interventions in primary care: a systematic review. *J Gen Intern Med.* 2015;30(1):107-117.
17. Lyzwinski LN. A systematic review and meta-analysis of mobile devices and weight loss with an intervention content analysis. *J Pers Med.* 2014;4(3):311-385.
18. Morris ME, Aguilera A. Mobile, Social, and Wearable Computing and the Evolution of Psychological Practice. *Prof Psychol Res Pr.* 2012;43(6):622-626.
19. Dennison L, Morrison L, Conway G, Yardley L. Opportunities and challenges for smartphone applications in supporting health behavior change: qualitative study. *J Med Internet Res.* 2013;15(4):e86.
