# Supplementary material for: Effect of a Smartphone App on Weight Change and Metabolic Outcomes in Asian Adults With Type 2 Diabetes: A Randomized Clinical Trial
Source: JAMA Netw Open. 2021 Jun 3;4(6):e2112417. doi: 10.1001/jamanetworkopen.2021.12417 (PMC8176331; doi:10.1001/jamanetworkopen.2021.12417)
Supplement: Supplement 3. — Data Sharing Statement [file jamanetwopen-e2112417-s003.pdf]

Lim SL, Ong KW, Johal J, et al. Effect of a smartphone app on weight change and metabolic outcomes in Asian adults with type 2 diabetes: a randomized clinical trial. *JAMA Netw Open*. 2021;4(6):e2112417. doi:10.1001/jamanetworkopen.2021.12417

Data Sharing Statement

Data

Data available: No

Additional Information

Explanation for why data not available: The dataset generated and/or analyzed for the purpose of the present study are not publicly available due to data confidentiality requirements of the ethics committee, but will be available from the corresponding author on reasonable request and approval from the ethics committee.
